# 6-Bromo-*N*-(6-bromo­pyridin-2-yl)-*N*-[4-(2,3-di­hydro­thieno[3,4-*b*][1,4]dioxin-5-yl)phen­yl]pyridin-2-amine

**DOI:** 10.1107/S1600536814013191

**Published:** 2014-06-21

**Authors:** Lauren A. Mitchell, Bradley J. Holliday

**Affiliations:** a Department of Chemistry, The University of Texas at Austin, 105 E 24th Street, Stop A5300, Austin, Texas 78712, USA

## Abstract

In the title mol­ecule, C_22_H_15_Br_2_N_3_O_2_S, the central benzene ring forms dihedral angles of 12.39 (17), 56.66 (17) and 74.71 (19)°, respectively, with the mean planes of the thio­phene and two pyridine rings. The dioxane ring is in a half-chair conformation. An intra­molecular C—H⋯O hydrogen forms an *S*(6) ring. The amine N atom is *sp* ^2^-hybridized.

## Related literature   

For related structures, see: Chen *et al.* (2011 ▶); Sotzing & Reynolds (1996 ▶); de Betterncourt-Dias *et al.* (2011 ▶). For applications of simliar compounds, see: Chahma *et al.* (2007 ▶); Roncali *et al.* (2005 ▶). For the synthesis of the starting material 4-(2,3-di­hydro­thieno[3,4-*b*][1,4]dioxin-5-yl)aniline, see: Trippé-Allard & Lacroix (2013 ▶). For the calculation of the functionality of the amine group in terms of hybridization, see: Allen *et al.* (1995 ▶). For hydrogen-bond graph-set motifs, see: Bernstein *et al.* (1995 ▶).
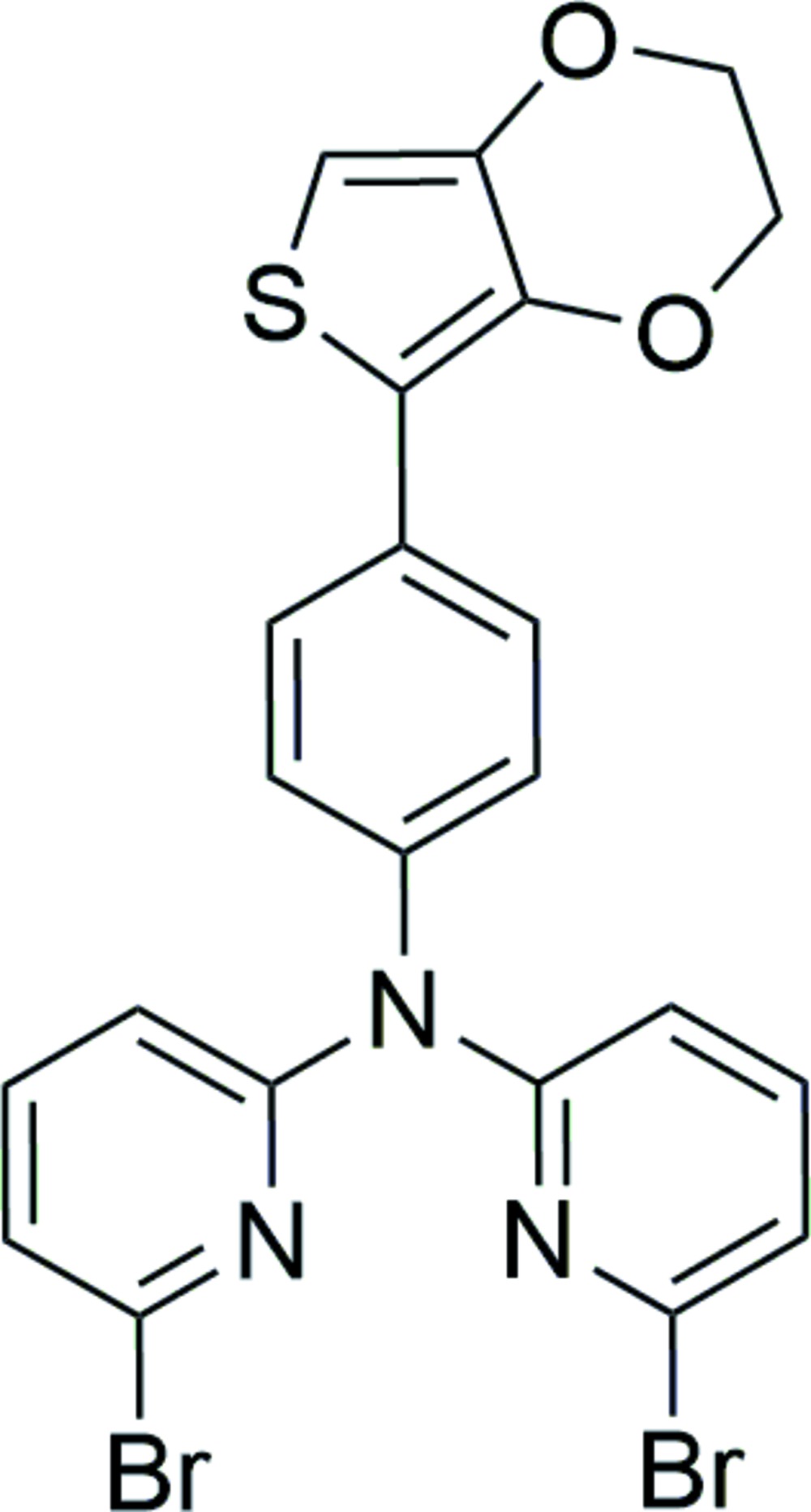


## Experimental   

### 

#### Crystal data  

C_22_H_15_Br_2_N_3_O_2_S
*M* *_r_* = 545.25
Triclinic, 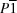

*a* = 4.483 (4) Å
*b* = 12.151 (9) Å
*c* = 18.958 (13) Å
α = 75.807 (18)°
β = 87.67 (3)°
γ = 89.62 (2)°
*V* = 1000.3 (13) Å^3^
*Z* = 2
Mo *K*α radiation
μ = 4.18 mm^−1^
*T* = 100 K
0.22 × 0.03 × 0.03 mm

#### Data collection  

Rigaku Saturn724+ diffractometer
Absorption correction: multi-scan (*ABSCOR*; Higashi, 2001 ▶) *T*_min_ = 0.563, *T*_max_ = 1.000
13439 measured reflections
3521 independent reflections
2732 reflections with *I* > 2σ(*I*)
*R*_int_ = 0.079

#### Refinement  

*R*[*F* ^2^ > 2σ(*F* ^2^)] = 0.051
*wR*(*F* ^2^) = 0.128
*S* = 1.00
3521 reflections
271 parameters
H-atom parameters constrained
Δρ_max_ = 1.06 e Å^−3^
Δρ_min_ = −0.83 e Å^−3^

### 

Data collection: *CrystalClear* (Rigaku, 2008 ▶); cell refinement: *CrystalClear*; data reduction: *CrystalClear*; program(s) used to solve structure: *SIR97* (Altomare *et al.*, 1999 ▶); program(s) used to refine structure: *SHELXL97* (Sheldrick, 2008 ▶) within *WinGX* (Farrugia, 2012 ▶); molecular graphics: *ORTEP-3 for Windows* (Farrugia, 2012 ▶) and *POV-RAY* (Cason, 2004 ▶); software used to prepare material for publication: *SHELXL97* and *publCIF* (Westrip, 2010 ▶).

## Supplementary Material

Crystal structure: contains datablock(s) I. DOI: 10.1107/S1600536814013191/lh5709sup1.cif

Structure factors: contains datablock(s) I. DOI: 10.1107/S1600536814013191/lh5709Isup2.hkl

Supporting information file. DOI: 10.1107/S1600536814013191/lh5709Isup3.cml

Additional supporting information:  crystallographic information; 3D view; checkCIF report

## Figures and Tables

**Table 1:** Hydrogen-bond geometry (Å, °)

*D*—H⋯*A*	*D*—H	H⋯*A*	*D*⋯*A*	*D*—H⋯*A*
C12—H12⋯O2	0.93	2.42	3.036 (7)	124

**Fig. 1.**
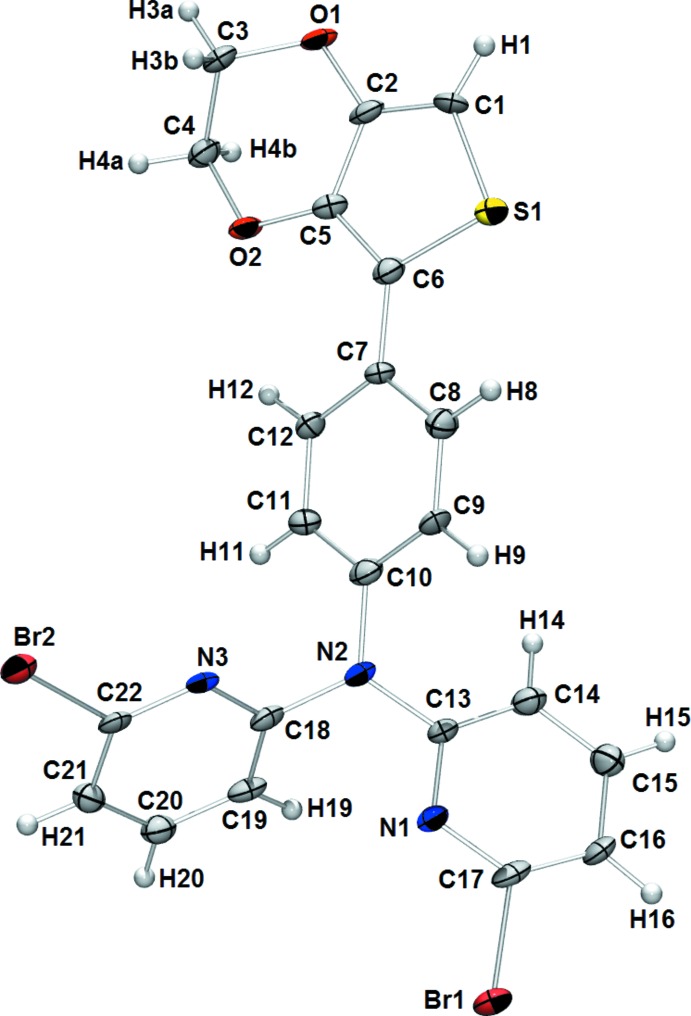
Molecular structure of the title compound. Ellipsoids are drawn at the 50% probability level.

## supplementary crystallographic information

### S1. Comment

The optical and electronic properties of 3,4-ethylenedioxythiophene (EDOT) containing compounds have spurred the development of materials for use in light-emitting devices, non-linear optics, and organic semi-conductors (Roncali *et al.*, 2005). Triphenylamines with EDOT substituants have been utilized in the development of electroactive polymers with high redox stabilities (Chahma *et al.*, 2007). The title compound is a promising precurser to branched unsymmetric electroactive polymers.

The geometry of the EDOT moiety is similar to other ethylenedioxythiophene containing compounds reported in the literature (Chen *et al.*, 2011; Sotzing & Reynolds, 1996). The dihedral angle between the thiophene and central benzene is 12.39 (17)°. The two pyridine rings are twisted out of plane of the benzene ring. The dihedral angle between the benzene ring and the pyridine ring containing N1 is 56.66 (17)°, and the dihedral angle between the benzene ring and the pyridine ring containing N2 is 74.71 (19)°. An intramolecular C—H···O hydrogen forms an S(6) ring (Bernstein *et al.*, 1995).

The pyramidality of the amine functionality, measured by χn, the angle between the C10—N2 vector and the N2/C13/C18 plane, described by Allen *et al.* (1995), is 2.3 (6)°, indicating that the hybridization of the nitrogen atom is mainly *sp^2^* (*sp^2^*χ_n_≈ 0°, *sp^3^*χ_n_≈ 60°).

### S2. Experimental

In an air-free glovebox *tris*(dibenzylideneacetone)dipalladium(0) (0.488 g, 0.5 mmol) was added to a dry schlenk flask. The reaction flask was pumped out, dry toluene was transferred into the flask by cannula and 4-(2,3-dihydrothieno[3,4-*b*][1,4]dioxin-5-yl)aniline, synthesized from Trippé-Allard & Lacroix (2013), (4.508 g, 19.3 mmol), 2,6-dibromopyridine (9.387 g, 39.6 mmol), 1,1'-*bis*(diphenylphosphino)ferrocene (0.632 g, 1.1 mmol), and sodium *tert*-butoxide (3.989 g, 41.5 mmol) were added to the solution. The solution was refluxed at 393 K for 20 h. The solution was cooled to room temperature and the toluene was removed by rotoevaporation. The product was extracted into CH_2_Cl_2_ (x3) washing with H_2_O. The crude solid was purified by silica gel column chromatography with 45% ethyl acetate: 55% hexanes by volume (*R*_f_ = 0.59) to yield a bright yellow solid (2.298 g, 21.8%). Crystals suitable for X-ray diffraction were obtained by slow evaporation from a 45% ethyl acetate, 55% hexanes solution (*v*/*v*). m.p. 433 K. ^1^H NMR (300 MHz, CDCl_3_) δ: 7.72 (d, 2H, *J* = 8.4), 7.36 (t, 2H, *J* = 7.9), 7.15 (d, 2H, *J* = 8.4), 7.09 (d, 2H, *J* = 5.1), 6.93 (d, 2H, *J* = 8.4), 6.30 (s, 1H), 4.31 – 4.25 (m, 4H), ^13^C{^1^H} NMR (75 MHz, CDCl_3_) δ: 156.9, 142.2, 141.5, 139.6, 139.4, 138.3, 131.51, 127.2, 122.1, 116.6, 114.9, 97.9, 64.8, 64.4. Anal. calcd. for C_22_H_15_Br_2_N_3_O_2_S: C, 48.46; H, 2.77; N, 7.71. Found: C, 48.63; H, 2.51; N, 7.59.

### S3. Refinement

All H atoms were positioned geometrically and refined using a riding model, with C—H = 0.93–0.97 Å and with *U*_iso_(H) = 1.2 times *U*_eq_(C).

### Crystal data

**Table:**

C_22_H_15_Br_2_N_3_O_2_S	*Z* = 2
*M**_r_* = 545.25	*F*(000) = 540
Triclinic, *P*1	*D*_x_ = 1.810 Mg m^−^^3^
Hall symbol: -P 1	Mo *K*α radiation, λ = 0.71075 Å
*a* = 4.483 (4) Å	Cell parameters from 3281 reflections
*b* = 12.151 (9) Å	θ = 1.7–27.7°
*c* = 18.958 (13) Å	µ = 4.18 mm^−^^1^
α = 75.807 (18)°	*T* = 100 K
β = 87.67 (3)°	Prism, colorless
γ = 89.62 (2)°	0.22 × 0.03 × 0.03 mm
*V* = 1000.3 (13) Å^3^	

### Data collection

**Table:**

Rigaku Saturn724+ diffractometer	3521 independent reflections
Radiation source: fine-focus sealed tube	2732 reflections with *I* > 2σ(*I*)
Graphite monochromator	*R*_int_ = 0.079
Detector resolution: 28.5714 pixels mm^-1^	θ_max_ = 25.0°, θ_min_ = 1.7°
dtprofit.ref scans	*h* = −5→5
Absorption correction: multi-scan (*ABSCOR*; Higashi, 2001)	*k* = −14→14
*T*_min_ = 0.563, *T*_max_ = 1.000	*l* = −22→22
13439 measured reflections	

### Refinement

**Table:**

Refinement on *F*^2^	Primary atom site location: structure-invariant direct methods
Least-squares matrix: full	Secondary atom site location: difference Fourier map
*R*[*F*^2^ > 2σ(*F*^2^)] = 0.051	Hydrogen site location: inferred from neighbouring sites
*wR*(*F*^2^) = 0.128	H-atom parameters constrained
*S* = 1.00	*w* = 1/[σ^2^(*F*_o_^2^) + (0.0638*P*)^2^] where *P* = (*F*_o_^2^ + 2*F*_c_^2^)/3
3521 reflections	(Δ/σ)_max_ = 0.001
271 parameters	Δρ_max_ = 1.06 e Å^−^^3^
0 restraints	Δρ_min_ = −0.83 e Å^−^^3^

### Special details

**Table:**

Geometry. All e.s.d.'s (except the e.s.d. in the dihedral angle between two l.s. planes) are estimated using the full covariance matrix. The cell e.s.d.'s are taken into account individually in the estimation of e.s.d.'s in distances, angles and torsion angles; correlations between e.s.d.'s in cell parameters are only used when they are defined by crystal symmetry. An approximate (isotropic) treatment of cell e.s.d.'s is used for estimating e.s.d.'s involving l.s. planes.
Refinement. Refinement of *F*^2^ against ALL reflections. The weighted *R*-factor *wR* and goodness of fit *S* are based on *F*^2^, conventional *R*-factors *R* are based on *F*, with *F* set to zero for negative *F*^2^. The threshold expression of *F*^2^ > σ(*F*^2^) is used only for calculating *R*-factors(gt) *etc*. and is not relevant to the choice of reflections for refinement. *R*-factors based on *F*^2^ are statistically about twice as large as those based on *F*, and *R*-factors based on ALL data will be even larger.

### Fractional atomic coordinates and isotropic or equivalent isotropic displacement parameters (Å^2^)

**Table:**

	*x*	*y*	*z*	*U*_iso_*/*U*_eq_	
C1	0.4149 (12)	0.8677 (5)	0.3436 (3)	0.0254 (13)	
H1	0.4019	0.9100	0.2958	0.030*	
C2	0.5672 (11)	0.7710 (5)	0.3635 (3)	0.0223 (12)	
C3	0.7454 (12)	0.6007 (5)	0.3458 (3)	0.0244 (13)	
H3A	0.8736	0.5675	0.3141	0.029*	
H3B	0.5491	0.5663	0.3489	0.029*	
C4	0.8723 (12)	0.5774 (5)	0.4210 (3)	0.0238 (12)	
H4A	0.8982	0.4962	0.4395	0.029*	
H4B	1.0666	0.6133	0.4177	0.029*	
C5	0.5503 (10)	0.7210 (5)	0.4403 (3)	0.0188 (11)	
C6	0.3812 (11)	0.7836 (5)	0.4784 (3)	0.0214 (12)	
C7	0.3032 (11)	0.7649 (4)	0.5558 (3)	0.0181 (11)	
C8	0.0814 (12)	0.8290 (5)	0.5805 (3)	0.0238 (12)	
H8	−0.0179	0.8841	0.5467	0.029*	
C9	0.0052 (12)	0.8131 (5)	0.6533 (3)	0.0235 (12)	
H9	−0.1470	0.8560	0.6680	0.028*	
C10	0.1546 (11)	0.7335 (5)	0.7049 (3)	0.0216 (12)	
C11	0.3738 (11)	0.6680 (5)	0.6820 (3)	0.0215 (12)	
H11	0.4733	0.6138	0.7163	0.026*	
C12	0.4468 (11)	0.6822 (5)	0.6089 (3)	0.0208 (12)	
H12	0.5927	0.6366	0.5945	0.025*	
C13	0.0144 (11)	0.8117 (5)	0.8102 (3)	0.0201 (12)	
C14	0.1319 (11)	0.9198 (5)	0.7798 (3)	0.0248 (13)	
H14	0.2632	0.9325	0.7393	0.030*	
C15	0.0499 (12)	1.0077 (5)	0.8109 (3)	0.0264 (13)	
H15	0.1216	1.0809	0.7910	0.032*	
C16	−0.1433 (12)	0.9848 (5)	0.8729 (3)	0.0257 (13)	
H16	−0.2045	1.0416	0.8954	0.031*	
C17	−0.2361 (11)	0.8761 (5)	0.8984 (3)	0.0235 (12)	
C18	0.0708 (11)	0.6057 (5)	0.8273 (3)	0.0204 (12)	
C19	0.2220 (12)	0.5796 (5)	0.8916 (3)	0.0230 (12)	
H19	0.3344	0.6341	0.9057	0.028*	
C20	0.2000 (11)	0.4706 (5)	0.9338 (3)	0.0239 (12)	
H20	0.2952	0.4508	0.9778	0.029*	
C21	0.0373 (11)	0.3899 (5)	0.9114 (3)	0.0221 (12)	
H21	0.0175	0.3157	0.9394	0.027*	
C22	−0.0947 (11)	0.4255 (5)	0.8448 (3)	0.0214 (12)	
N1	−0.1728 (9)	0.7893 (4)	0.8702 (2)	0.0208 (10)	
N2	0.0834 (10)	0.7185 (4)	0.7811 (2)	0.0223 (10)	
N3	−0.0829 (9)	0.5300 (4)	0.8032 (2)	0.0208 (10)	
O1	0.7239 (8)	0.7215 (3)	0.31571 (18)	0.0250 (9)	
O2	0.6761 (8)	0.6202 (3)	0.47078 (18)	0.0228 (9)	
S1	0.2461 (3)	0.90420 (13)	0.41770 (7)	0.0266 (3)	
Br1	−0.49638 (12)	0.83856 (5)	0.98380 (3)	0.02763 (19)	
Br2	−0.31232 (12)	0.31910 (5)	0.80909 (3)	0.02536 (18)	

### Atomic displacement parameters (Å^2^)

**Table:**

	*U*^11^	*U*^22^	*U*^33^	*U*^12^	*U*^13^	*U*^23^
C1	0.042 (3)	0.024 (3)	0.009 (3)	0.000 (3)	0.005 (2)	−0.002 (2)
C2	0.028 (3)	0.026 (3)	0.017 (3)	−0.004 (2)	0.004 (2)	−0.014 (2)
C3	0.033 (3)	0.025 (3)	0.019 (3)	0.002 (2)	0.001 (2)	−0.014 (3)
C4	0.028 (3)	0.025 (3)	0.022 (3)	0.002 (2)	0.001 (2)	−0.012 (2)
C5	0.018 (3)	0.026 (3)	0.015 (3)	0.000 (2)	0.0007 (19)	−0.009 (2)
C6	0.026 (3)	0.023 (3)	0.018 (3)	0.002 (2)	−0.002 (2)	−0.010 (2)
C7	0.025 (3)	0.019 (3)	0.011 (2)	0.001 (2)	0.001 (2)	−0.006 (2)
C8	0.029 (3)	0.024 (3)	0.019 (3)	0.007 (2)	−0.003 (2)	−0.006 (2)
C9	0.032 (3)	0.021 (3)	0.018 (3)	0.004 (2)	0.009 (2)	−0.009 (2)
C10	0.026 (3)	0.024 (3)	0.018 (3)	−0.003 (2)	0.001 (2)	−0.011 (2)
C11	0.029 (3)	0.022 (3)	0.015 (3)	0.000 (2)	−0.001 (2)	−0.006 (2)
C12	0.025 (3)	0.020 (3)	0.018 (3)	0.004 (2)	0.003 (2)	−0.007 (2)
C13	0.028 (3)	0.019 (3)	0.014 (3)	0.001 (2)	0.001 (2)	−0.008 (2)
C14	0.029 (3)	0.027 (3)	0.020 (3)	−0.001 (2)	0.005 (2)	−0.010 (3)
C15	0.033 (3)	0.024 (3)	0.022 (3)	−0.005 (2)	0.002 (2)	−0.006 (2)
C16	0.038 (3)	0.023 (3)	0.021 (3)	0.001 (3)	0.004 (2)	−0.015 (3)
C17	0.025 (3)	0.033 (4)	0.017 (3)	0.006 (2)	−0.003 (2)	−0.016 (3)
C18	0.022 (3)	0.025 (3)	0.018 (3)	0.005 (2)	0.001 (2)	−0.013 (2)
C19	0.024 (3)	0.033 (3)	0.016 (3)	0.003 (2)	−0.001 (2)	−0.014 (3)
C20	0.028 (3)	0.027 (3)	0.018 (3)	0.003 (2)	−0.001 (2)	−0.008 (3)
C21	0.025 (3)	0.022 (3)	0.018 (3)	0.002 (2)	0.001 (2)	−0.003 (2)
C22	0.029 (3)	0.023 (3)	0.015 (3)	−0.001 (2)	0.008 (2)	−0.011 (2)
N1	0.027 (2)	0.021 (3)	0.016 (2)	0.0047 (19)	−0.0002 (18)	−0.009 (2)
N2	0.032 (2)	0.021 (3)	0.017 (2)	−0.001 (2)	0.0055 (18)	−0.011 (2)
N3	0.029 (2)	0.022 (3)	0.013 (2)	0.000 (2)	0.0068 (18)	−0.009 (2)
O1	0.035 (2)	0.030 (2)	0.0138 (18)	−0.0010 (17)	0.0057 (15)	−0.0128 (17)
O2	0.031 (2)	0.027 (2)	0.0130 (18)	0.0098 (17)	0.0015 (15)	−0.0090 (17)
S1	0.0396 (8)	0.0243 (8)	0.0158 (7)	0.0065 (6)	0.0026 (6)	−0.0057 (6)
Br1	0.0357 (3)	0.0294 (4)	0.0196 (3)	0.0038 (3)	0.0076 (2)	−0.0111 (3)
Br2	0.0329 (3)	0.0260 (4)	0.0200 (3)	−0.0033 (2)	0.0029 (2)	−0.0115 (2)

### Geometric parameters (Å, º)

**Table:**

C1—C2	1.335 (7)	C11—H11	0.9300
C1—S1	1.719 (5)	C12—H12	0.9300
C1—H1	0.9300	C13—N1	1.360 (6)
C2—O1	1.374 (6)	C13—C14	1.396 (8)
C2—C5	1.433 (7)	C13—N2	1.403 (7)
C3—O1	1.442 (6)	C14—C15	1.382 (8)
C3—C4	1.517 (7)	C14—H14	0.9300
C3—H3A	0.9700	C15—C16	1.403 (7)
C3—H3B	0.9700	C15—H15	0.9300
C4—O2	1.450 (6)	C16—C17	1.353 (8)
C4—H4A	0.9700	C16—H16	0.9300
C4—H4B	0.9700	C17—N1	1.318 (7)
C5—O2	1.351 (6)	C17—Br1	1.919 (5)
C5—C6	1.376 (7)	C18—N3	1.330 (7)
C6—C7	1.457 (7)	C18—C19	1.386 (7)
C6—S1	1.748 (5)	C18—N2	1.435 (7)
C7—C8	1.394 (7)	C19—C20	1.371 (8)
C7—C12	1.409 (7)	C19—H19	0.9300
C8—C9	1.375 (7)	C20—C21	1.384 (8)
C8—H8	0.9300	C20—H20	0.9300
C9—C10	1.386 (7)	C21—C22	1.386 (7)
C9—H9	0.9300	C21—H21	0.9300
C10—C11	1.382 (7)	C22—N3	1.319 (7)
C10—N2	1.433 (6)	C22—Br2	1.893 (6)
C11—C12	1.380 (7)		
			
C2—C1—S1	111.4 (4)	C11—C12—H12	119.6
C2—C1—H1	124.3	C7—C12—H12	119.6
S1—C1—H1	124.3	N1—C13—C14	122.3 (5)
C1—C2—O1	124.1 (5)	N1—C13—N2	115.4 (5)
C1—C2—C5	113.8 (5)	C14—C13—N2	122.3 (5)
O1—C2—C5	122.1 (5)	C15—C14—C13	118.9 (5)
O1—C3—C4	109.9 (4)	C15—C14—H14	120.6
O1—C3—H3A	109.7	C13—C14—H14	120.6
C4—C3—H3A	109.7	C14—C15—C16	118.9 (6)
O1—C3—H3B	109.7	C14—C15—H15	120.5
C4—C3—H3B	109.7	C16—C15—H15	120.5
H3A—C3—H3B	108.2	C17—C16—C15	116.7 (5)
O2—C4—C3	111.0 (4)	C17—C16—H16	121.6
O2—C4—H4A	109.4	C15—C16—H16	121.6
C3—C4—H4A	109.4	N1—C17—C16	127.3 (5)
O2—C4—H4B	109.4	N1—C17—Br1	113.8 (4)
C3—C4—H4B	109.4	C16—C17—Br1	118.9 (4)
H4A—C4—H4B	108.0	N3—C18—C19	123.6 (5)
O2—C5—C6	124.2 (5)	N3—C18—N2	116.0 (4)
O2—C5—C2	122.8 (4)	C19—C18—N2	120.3 (5)
C6—C5—C2	112.9 (5)	C20—C19—C18	117.6 (5)
C5—C6—C7	131.5 (5)	C20—C19—H19	121.2
C5—C6—S1	109.3 (4)	C18—C19—H19	121.2
C7—C6—S1	119.2 (4)	C19—C20—C21	120.5 (5)
C8—C7—C12	117.1 (5)	C19—C20—H20	119.8
C8—C7—C6	120.9 (4)	C21—C20—H20	119.8
C12—C7—C6	122.0 (4)	C20—C21—C22	116.3 (5)
C9—C8—C7	121.9 (5)	C20—C21—H21	121.8
C9—C8—H8	119.0	C22—C21—H21	121.8
C7—C8—H8	119.0	N3—C22—C21	125.0 (5)
C8—C9—C10	120.2 (5)	N3—C22—Br2	116.1 (4)
C8—C9—H9	119.9	C21—C22—Br2	118.9 (4)
C10—C9—H9	119.9	C17—N1—C13	115.8 (5)
C11—C10—C9	119.1 (5)	C13—N2—C10	121.0 (4)
C11—C10—N2	120.1 (5)	C13—N2—C18	119.9 (4)
C9—C10—N2	120.8 (5)	C10—N2—C18	119.0 (4)
C12—C11—C10	120.8 (5)	C22—N3—C18	116.9 (4)
C12—C11—H11	119.6	C2—O1—C3	110.1 (4)
C10—C11—H11	119.6	C5—O2—C4	113.7 (4)
C11—C12—C7	120.9 (5)	C1—S1—C6	92.6 (3)
			
S1—C1—C2—O1	179.9 (4)	C18—C19—C20—C21	1.4 (8)
S1—C1—C2—C5	0.8 (6)	C19—C20—C21—C22	0.8 (7)
O1—C3—C4—O2	62.8 (6)	C20—C21—C22—N3	−2.0 (8)
C1—C2—C5—O2	176.4 (5)	C20—C21—C22—Br2	178.4 (4)
O1—C2—C5—O2	−2.7 (8)	C16—C17—N1—C13	2.2 (8)
C1—C2—C5—C6	−0.5 (7)	Br1—C17—N1—C13	−179.1 (3)
O1—C2—C5—C6	−179.6 (5)	C14—C13—N1—C17	−0.3 (7)
O2—C5—C6—C7	2.6 (9)	N2—C13—N1—C17	179.3 (4)
C2—C5—C6—C7	179.5 (5)	N1—C13—N2—C10	151.1 (5)
O2—C5—C6—S1	−176.9 (4)	C14—C13—N2—C10	−29.4 (7)
C2—C5—C6—S1	−0.1 (6)	N1—C13—N2—C18	−26.2 (7)
C5—C6—C7—C8	−167.6 (6)	C14—C13—N2—C18	153.3 (5)
S1—C6—C7—C8	11.9 (7)	C11—C10—N2—C13	142.0 (5)
C5—C6—C7—C12	12.5 (9)	C9—C10—N2—C13	−38.1 (7)
S1—C6—C7—C12	−168.0 (4)	C11—C10—N2—C18	−40.6 (7)
C12—C7—C8—C9	0.2 (8)	C9—C10—N2—C18	139.2 (5)
C6—C7—C8—C9	−179.7 (5)	N3—C18—N2—C13	130.1 (5)
C7—C8—C9—C10	1.5 (9)	C19—C18—N2—C13	−52.2 (7)
C8—C9—C10—C11	−2.0 (9)	N3—C18—N2—C10	−47.3 (6)
C8—C9—C10—N2	178.1 (5)	C19—C18—N2—C10	130.4 (5)
C9—C10—C11—C12	0.8 (9)	C21—C22—N3—C18	0.8 (7)
N2—C10—C11—C12	−179.4 (5)	Br2—C22—N3—C18	−179.6 (3)
C10—C11—C12—C7	1.0 (9)	C19—C18—N3—C22	1.7 (7)
C8—C7—C12—C11	−1.5 (8)	N2—C18—N3—C22	179.4 (4)
C6—C7—C12—C11	178.4 (5)	C1—C2—O1—C3	−153.8 (5)
N1—C13—C14—C15	−1.5 (8)	C5—C2—O1—C3	25.2 (7)
N2—C13—C14—C15	179.0 (5)	C4—C3—O1—C2	−53.4 (5)
C13—C14—C15—C16	1.4 (8)	C6—C5—O2—C4	−173.1 (5)
C14—C15—C16—C17	0.2 (8)	C2—C5—O2—C4	10.3 (7)
C15—C16—C17—N1	−2.3 (9)	C3—C4—O2—C5	−39.1 (6)
C15—C16—C17—Br1	179.2 (4)	C2—C1—S1—C6	−0.7 (5)
N3—C18—C19—C20	−2.8 (8)	C5—C6—S1—C1	0.4 (4)
N2—C18—C19—C20	179.6 (4)	C7—C6—S1—C1	−179.2 (5)

### Hydrogen-bond geometry (Å, º)

**Table:**

*D*—H···*A*	*D*—H	H···*A*	*D*···*A*	*D*—H···*A*
C12—H12···O2	0.93	2.42	3.036 (7)	124
